# Salinomycin-Loaded Iron Oxide Nanoparticles for Glioblastoma Therapy

**DOI:** 10.3390/nano10030477

**Published:** 2020-03-06

**Authors:** Mohammad Norouzi, Vinith Yathindranath, James A. Thliveris, Donald W. Miller

**Affiliations:** 1 Department of Biomedical Engineering, University of Manitoba, Winnipeg, MB R3T 5V6, Canada; norouzim@myumanitoba.ca; 2 Department of Pharmacology and Therapeutics, University of Manitoba, Winnipeg, MB R3E 0Z3, Canada; Vinith.Yathindranath@umanitoba.ca; 3 Department of Human Anatomy and Cell Science University of Manitoba, Winnipeg, MB R3E 0J9, Canada; james.thliveris@gmail.com

**Keywords:** iron oxide nanoparticles, salinomycin, GBM, drug delivery, blood–brain barrier, external magnetic field

## Abstract

Salinomycin is an antibiotic introduced recently as a new and effective anticancer drug. In this study, magnetic iron oxide nanoparticles (IONPs) were utilized as a drug carrier for salinomycin for potential use in glioblastoma (GBM) chemotherapy. The biocompatible polyethylenimine (PEI)-polyethylene glycol (PEG)-IONPs (PEI-PEG-IONPs) exhibited an efficient uptake in both mouse brain-derived microvessel endothelial (bEnd.3) and human U251 GBM cell lines. The salinomycin (Sali)-loaded PEI-PEG-IONPs (Sali-PEI-PEG-IONPs) released salinomycin over 4 days, with an initial release of 44% ± 3% that increased to 66% ± 5% in acidic pH. The Sali-IONPs inhibited U251 cell proliferation and decreased their viability (by approximately 70% within 48 h), and the nanoparticles were found to be effective in reactive oxygen species-mediated GBM cell death. Gene studies revealed significant activation of caspases in U251 cells upon treatment with Sali-IONPs. Furthermore, the upregulation of tumor suppressors (i.e., p53, Rbl2, Gas5) was observed, while TopII, Ku70, CyclinD1, and Wnt1 were concomitantly downregulated. When examined in an *in vitro* blood–brain barrier (BBB)-GBM co-culture model, Sali-IONPs had limited penetration (1.0% ± 0.08%) through the bEnd.3 monolayer and resulted in 60% viability of U251 cells. However, hyperosmotic disruption coupled with an applied external magnetic field significantly enhanced the permeability of Sali-IONPs across bEnd.3 monolayers (3.2% ± 0.1%) and reduced the viability of U251 cells to 38%. These findings suggest that Sali-IONPs combined with penetration enhancers, such as hyperosmotic mannitol and external magnetic fields, can potentially provide effective and site-specific magnetic targeting for GBM chemotherapy.

## 1. Introduction

Glioblastoma multiforme (GBM) is the most prevalent and aggressive form of primary brain tumors in adults, the current standard of care for which includes surgical recession of tumor followed by radio- and chemotherapies [1,2,3]. However, the extensive infiltrative nature of GBM tumors makes complete surgical recession difficult. Furthermore, the presence of the blood–brain barrier (BBB) limits the penetration of many chemotherapeutics into the brain and poses a significant obstacle in an effective treatment of GBM [4,5,6]. The BBB is composed of a continuous endothelium surrounded by astrocytic foot processes and pericytes that together regulate the passage of substances from the bloodstream into the brain [7]. In addition to the tight junctions, brain endothelial cells also express a number of efflux transporters, such as P-glycoprotein (P-gp) and breast cancer resistance protein (BCRP), limiting drug penetration into the brain [8]. In this regard, most of the current chemotherapeutics suffer from an inability to penetrate the BBB effectively, resulting in limited therapeutic effects. Furthermore, simply increasing the dose of chemotherapeutics administered to achieve the desired therapeutic concentration at the tumor site is not possible due to systemic adverse side effects [7,9].

To circumvent these hurdles, a variety of engineered nanoparticles (e.g., gold nanoparticles, nanoliposomes) have been developed as drug delivery systems, with the capability of transporting therapeutic agents across the BBB and targeting GBM cells [10,11,12,13]. Amidst the broad range of engineered nanoparticles, iron oxide nanoparticles (IONPs, magnetite (Fe_3_O_4_) or maghemite (γ-Fe_2_O_3_)) offer many advantages in cancer theranostics by virtue of their tunable, size-dependent magnetic properties [7,14]. The inherent magnetic properties of the IONPs not only make them ideal candidates as contrast agents for magnetic resonance imaging (MRI), but also for site-specific magnetic targeting, utilizing an external magnetic field. In addition, among various metal oxide nanoparticles, IONPs coated with biocompatible polymers have shown a good safety profile, with the components being shuttled into the body’s iron cycle upon degradation [7,14,15]. For these reasons, IONPs have emerged as potential nanocarriers for site-specific magnetic targeting of anticancer drugs, antibodies, peptides, and small interfering RNA [7,14,16,17].

Thus far, IONPs have been developed as efficacious nanocarriers for a variety of anticancer drugs in glioma therapy, including doxorubicin [18], paclitaxel [19], gemcitabine [20], cetuximab [21], and EGFRvIIIAb [22]. Moreover, several IONPs have been developed as MRI contrast agents in clinical trials, such as ferumoxide (Feridex^®^), ferumoxytol (Feraheme^®^), ferucarbotran (Resovist^®^), and ferumoxtran-10 (Combidex^®^), owing to their effective reduction of T1, T2, and T2* relaxation times [23,24]. Although IONPs as drug carriers have not entered clinical trials yet, their utility in site-specific and enhanced drug delivery of chemotherapeutics have been widely reported in preclinical studies for treatment of malignant gliomas [11,25]. 

Salinomycin is an antibacterial, ionophore, and anticoccidial therapeutic drug whose anticancer effect has recently been identified [26,27]. As a chemotherapeutic, salinomycin was reported to be 100-fold more effective than paclitaxel in inducing apoptosis in breast cancer stem-like cells [28]. More recent studies have authenticated the anticancer effects of salinomycin on gastrointestinal sarcoma, osteosarcoma, and colorectal cancer [29]. Whereas the molecular mechanisms of salinomycin cytotoxicity have not been fully described, the release of cytochrome c and the activation of caspases are believed to be implicated in salinomycin’s anticancer mechanisms [29]. In addition, salinomycin can target cancer stem cells and prevent the Wnt/β-catenin pathway, which is crucial for stem cell self-renewal [27]. Furthermore, salinomycin may cause strong and time-dependent ATP-depletion in cancer cells [29] and interferes with potassium channels, promoting the efflux of potassium ions from mitochondria and cytoplasm, thus promoting cell apoptosis [30]. 

The anticancer effect of salinomycin-loaded poly (lactic-co-glycolic acid) (PLGA) nanofibers [31], as implantable drug carriers at the tumor cavity after surgical resection, has previously been reported on GBM cells, suggesting a potential application in local treatment of brain tumors. However, systemically administered salinomycin is not able to penetrate the BBB, and has reduced oral absorption because of the multidrug efflux transporter P-gp [32]. Therefore, development of intravenous (i.v.) drug delivery systems for salinomycin with the capability of crossing the BBB and entering the brain is of significant clinical importance.

In this study, surface-modified IONPs were synthesized and characterized as a potential delivery system for salinomycin. For this purpose, salinomycin (Sali)-loaded polyethylenimine (PEI) polyethylene glycol (PEG) IONPs (Sali-PEI-PEG-IONPs) were fabricated and their anticancer effects on GBM cells were investigated. Moreover, the permeability of the nanoparticle formulation across an *in vitro* model of the BBB was examined. 

## 2. Materials and Methods

### 2.1. Materials

All chemical reagents were purchased from Sigma-Aldrich (St. Louis, MO, USA), and all cell culture and biochemical reagents were obtained from Thermo Fisher Scientific, Inc. (Rockford, IL, USA), unless otherwise specified.

### 2.2. Synthesis and Characterization of IONPs

IONPs were synthesized as previously reported by our group [33]. Briefly, to synthesize IONP-Sil(NH_2_), Fe(acac)_3_ (2.83 g, 8 mmol) was dissolved in 6:4 ethanol/deionized water and purged with nitrogen for 1 h, followed by addition of NaBH_4_ (3.03 g, 80.0 mmol) in deoxygenated DI water under stirring (1000 rpm). After 20 min, the color of the reaction mixture changed from red to black, evincing the formation of IONPs. After 1 h, (3-aminopropyl) triethoxysilane (APTES, 16 mL, 17 mmol) was added, and the reaction mixture was stirred overnight at room temperature. The blackish-brown solution was filtered, and the solvent was removed at 50 °C under low pressure. The obtained viscous mixture was dissolved in 200 mL of cold ethanol and left until excess NaBH_4_ became crystallized, which was removed by filtration. This step was repeated until no further crystal was observed. Then, ethanol was completely evaporated, and the product was dissolved in 50 mL DI water and dialyzed (Spectra/Por MWCO 6-8000 dialysis membrane) against DI water to remove the unreacted APTES. The resulting mixture was centrifuged at 4000 rpm for 30 min and the dark reddish-brown supernatant (containing IONPs) was collected and stored for further use.

For the synthesis of PEI-PEG-IONPs, PEG diacid 600 (2.0 g, 3.3 mmol), 1-ethyl-3-(3-dimethylaminopropyl) carbodiimide (EDC, 0.19 g, 1 mmol), and N-hydroxysulfosuccinimide sodium salt (NHS, 0.21 g, 1 mmol) were dissolved in DI water and stirred for 15 min. Then, IONP-Sil(NH_2_) solution (∼42.0 mg of aminosilane, ∼0.3 mmol) was added to the mixture and stirred for an additional 3 h. The product was dialyzed against DI water followed by centrifugation at 4000 rpm. The obtained supernatant was collected and stored for further use. To accomplish the PEI coating, Na_2_CO_3_, NaHCO_3_ (Na_2_CO_3_ = 0.21198 g, NaHCO_3_ = 1.512 g), EDC (0.19 g, 1 mmol), NHS (0.21 g, 1 mmol), and IONP-PEG(COOH) were dissolved in 20 mL DI water under stirring. After 15 min, PEI (Mw: 2 kDa, 2 mg/mL) in 30 mL of DI water was added rapidly to the reaction mixture and mixed overnight. The following day, the obtained crude product was washed with DI water and dialyzed against DI water to yield PEI-PEG-IONPs.

Initial characterization of the PEI-PEG-IONP intermediates for physicochemical and magnetic properties has been previously reported [33]. The molar ratio of the coatings on IONPs was determined using thermogravimetric analysis (TGA), as described elsewhere [33]. For confirmation of the size and polydispersity of the PEI-PEG-IONPs, the IONP size distribution in DI water (pH 7.4) was determined by dynamic light scattering (DLS) measurements using a Photocor Complex system. The Fourier transform infrared (FTIR) spectrum was taken using a Thermo Nicolet iS10 FTIR spectrometer. Transmission electron microscope (TEM) images of the nanoparticles were acquired using a Philips CM 10 electron microscope (Hillsboro, OR, USA) to measure the core diameter of the PEI-PEG-IONPs. The diameter was measured for 100 nanoparticles from several TEM images, which were representative of the whole batch, using Image J software (1.48 v, National Institutes of Health, Bethesda, MD, USA). 

### 2.3. Drug Loading on IONPs

To load salinomycin on the synthesized PEI-PEG-IONPs, equal concentrations (30 µg/mL) of PEI-PEG-IONPs and salinomycin were mixed in phosphate-buffered saline (PBS, pH 6) and the reaction mixture was incubated overnight. The resulting mixture was then centrifuged at 12,000 rpm for 10 min and the aqueous supernatant was carefully removed. Subsequently, the salinomycin-loaded PEI-PEG-IONPs (Sali-PEI-PEG-IONPs) were re-suspended, washed twice with PBS (pH 7.4), and separated by centrifugation as described above to remove any non-adherent salinomycin.

### 2.4. Biocompatibility Evaluation of IONPs 

To evaluate the biocompatibility of the synthesized PEI-PEG-IONPs, a mouse brain-derived microvessel endothelial cell line, bEnd.3 (American Type Tissue Culture Collection, Manassas, VA, USA), was used as a cell culture model for the BBB, in addition to the authenticated human U251 GBM cell line. The bEnd.3 cells (passage number 20–30) were cultured in Dulbecco’s modified essential medium (DMEM, Gibco, UK), supplemented with 10% fetal bovine serum (FBS, Hyclone, Logan, UT) and 1% mL penicillin and streptomycin Invitrogen, USA). The U251 cells (passage number 20–30) were cultured in DMEM/F12 (Gibco, UK), supplemented with 10% FBS and 1% penicillin-streptomycin. To assess the effect of PEI-PEG-IONPs on cell viability, the bEnd.3 and U251 cells were seeded at a density of 2 × 10^4^ and 1 × 10^4^ cell/cm^2^, respectively, in 96-well plates, and incubated overnight at 37 °C to allow them to attach. Then, the cells were treated with PEI-PEG-IONPs at the concentrations of 0.25 to 50 µg/mL and suspended in the cell culture media for 48 h. Afterward, the culture media was removed, and the cells were washed with PBS and incubated in medium supplemented with 0.5 mg/mL 3-(4,5-dimethylthiazol-2-yl)-2,5-diphenyltetrazoliumbromide (MTT) reagent for 3 h at 37 °C. Then, the media was removed and the cells were solubilized in dimethyl sulfoxide (DMSO) [31]. The absorbance of the solubilized cells was determined using a Synergy HT plate reader (BioTek, Winooski, VT) at a wavelength of 570 nm. The relative cell viability was calculated using optical density (OD) measurement as [OD]_test_/[OD]_control_, and the average value was obtained from five replications.

### 2.5. Drug Release from IONPs

The release kinetics of salinomycin from the PEI-PEG-IONPs was determined at 37 °C in PBS (pH 7.4 to mimic pH of the blood and extracellular fluid, and pH 4.5 to mimic acidic tumor micro-environment and endosomal compartments). To this end, the Sali-PEI-PEG-IONPs (30 µg/mL) were suspended in 1 mL PBS. At various time points, the tubes were centrifuged at 12,000 rpm for 10 min to pellet the nanoparticles and the solution was entirely collected and replaced with 1 mL of fresh PBS. The concentration of the released salinomycin was measured using an Ionophore ELISA kit (Europroxima, The Netherlands), in compliance with the manufacturer’s protocol. Quantitative determinations of salinomycin released from the IONP as a function of incubation time were determined based on standard curves performed with each analysis.

### 2.6. Cellular Uptake of IONPs

Confluent monolayers of bEnd.3 and U251 cells were grown in 24-well culture plates and treated with culture media containing either PEI-PEG-IONPs or Sali-PEI-PEG-IONPs (30 µg/mL) for 4 h at 37 °C in both the presence and absence of a static external magnetic field. Afterward, the cell monolayers were washed 3 times with cold PBS to remove unbound nanoparticles, followed by lysing the cells with 0.1% Triton solution in PBS overnight at −20 °C. The IONP content was determined based on the ferrozine assay, as previously reported [34]. Briefly, 500 µL of 12 M HCl was added to each well and incubated for 1 h at room temperature with gentle shaking to solubilize the nanoparticles. The samples were then neutralized with 500 µL of 12 M NaOH, followed by the addition of 120 µL of hydroxylamine hydrochloride (2.8 M) in 4 M HCl and incubation at room temperature with gentle shaking for 1 h. Thereafter, 50 µL of 10 M ammonium acetate solution (pH 9.5) and 300 µL of 10 mM ferrozine in 0.1 M ammonium acetate solution were added consecutively to each sample, and the absorbance was measured at 562 nm using a Synergy HT plate reader. Quantitative determination of IONP concentration was fulfilled based on a standard curve prepared using various dilutions of an iron chloride atomic absorption standard (Fisher Scientific, Ottawa, ON). The protein content of the lysed cells was determined using a BCA protein assay kit (Pierce™ BCA protein assay kit, Thermo Fisher Scientific, Rockford, IL, USA).

The cellular localization of the PEI-PEG-IONPs was also examined using transmission electron microscopy (TEM). For this study, U251 cells were treated with the nanoparticles as described above. After 4 h, the media was removed, the cells were washed with PBS, and then disassociated using a 0.25% trypsin-ethylenediaminetetraacetic acid (EDTA) solution (Hyclone, Logan, UT, USA). The collected cells were then centrifuged (5 min at 1500 g) and the cell pellet was resuspended in 3% glutaraldehyde in 0.1 M phosphate buffer (pH 7.3) at room temperature for 3 h. This was followed by postfixation for 2 h at room temperature in 1% osmium tetroxide in 0.1 M phosphate buffer, dehydration in ascending concentrations of ethanol, and embedding in Epon resin. Thin sections were stained with uranyl acetate and lead citrate, visualized, and photographed by TEM. 

### 2.7. Cytotoxicity of Sali-IONPs in GBM Cell Line

The cytotoxicity of Sali-PEI-PEG-IONPs against U251 cells was evaluated using MTT assay. For this purpose, the cells were cultured as described previously. After a 24 h period, the media were changed with fresh media (negative control), media containing an equivalent amount of salinomycin corresponding to salinomycin released from Sali-PEI-PEG-IONPs over the same time (positive control, 1 µg/mL), PEI-PEG-IONPs, and Sali-PEI-PEG-IONPs. Following a 48 h treatment, viability of the cells was determined by MTT assay, as described in Section 2.4.

Moreover, to observe the effects of the treatments on the cell proliferation, the cells were labeled with 50 mM of fluorescent dye carboxyfluorescein succinimidyl ester (CFSE) for 20 min at 37 °C. The cellular content of CFSE is reduced during each cell division, resulting in a sequential halving of the cellular fluorescent intensity with each mitotic event [35]. Following the loading of CFSE into the cells, the media was changed, and the cells were washed and treated with either PEI-PEG-IONPs, salinomycin (1 µg/mL), or Sali-PEI-PEG-IONPs (equivalent to 1 µg/mL of salinomycin) for an additional 48 h. Following the 48 h exposure, the media was changed and the cells were left for 24 h without any further treatment. Thereafter, the fluorescence intensity of the cells was measured using flow cytometry (BD FACSCanto II Flow Cytometer instrument (BD Bioscience)). In addition, cell apoptosis was determined using a fluorescein isothiocyanate (FITC) Annexin V/ propidium iodide (PI) apoptosis Kit (Thermo Fisher Scientific, USA). For this purpose, the cells were treated similarly either with PEI-PEG-IONPs, salinomycin, or Sali-PEI-PEG-IONPs for 48 h, followed by 24 h incubation without any further treatment. Thereafter, the cells were stained with FITC Annexin V and PI according to the manufacturer’s protocol, and subsequently were analyzed using flow cytometry.

Morphology of the U251 cells after 48 h treatment was also studied using a fluorescence microscope (Zeiss Axio observer Z1, Germany). To this end, the treated cells were washed with PBS and fixed with 4% paraformaldehyde for 20 min at room temperature, followed by permeabilization with 0.2% Triton X-100 for 5 min. Then, actin cytoskeleton was stained with ActinRed for 30 min and the nucleus was stained with 4′,6-diamidino-2-phenylindole (DAPI) solution (100 nM) for 5 min at 37 °C. Afterwards, the samples were washed with PBS and visualized using the microscope.

### 2.8. Reactive Oxygen Species Determination

Intracellular reactive oxygen species (ROS) was measured based on the peroxide-dependent oxidation of the non-fluorescent 2’,7’-dichlorofuorescein diacetate (DCFDA). Upon entering the cells, acetate groups of the DCFDA are cleaved by intracellular esterases, being transformed to the highly fluorescent and cell impermeable 2’,7’-dichlorofluorescein (DCF) [36]. For this study, U251 cells were seeded in black 96-well plates at a density of 5000 cell/cm^2^ and cultured overnight. After washing the cells with PBS, they were stained with 50 μM DCFDA in PBS for 45 min at 37 °C. Then, the DCFDA solution was removed and the washed cells were treated with either PEI-PEG-IONPs, salinomycin, or Sali-PEI-PEG-IONPs in cell culture media for up to 72 h. The cellular accumulation of ROS in response to the treatments was calculated by measuring the oxidation of DCFDA to the fluorescent DCF using a Synergy HT fluorescent plate reader at Ex/Em = 485/535 nm.

### 2.9. Quantitative RT-PCR

The U251 cells were treated with either PEI-PEG-IONPs, salinomycin, or Sali-PEI-PEG-IONPs for 48 h. After washing the cells with PBS, the total RNA was extracted, utilizing TRIzol reagent (Invitrogen, Carlsbad, CA, USA) in accordance with the manufacturer’s protocol. The RNA’s purity and concentration were measured by UV-VIS spectrophotometry (NanoDrop, Thermo Fisher Scientific Inc, USA). Thenceforth, the expression levels of mRNA encoding Top II, Ku70, p53, caspase 9, caspase 3, cyclin D, Wnt 1, Rbl2, GAS5, and MIR155 was determined by quantitative reverse-transcript polymerase chain reaction (qRT-PCR). The RT-PCR was carried out using iTaq Universal SYBR Green supermix kit (Bio-Rad, Hercules, CA, USA) and β-actin was considered as the housekeeping gene. The reactions were conducted in an Applied Biosystems 7300 Real-Time PCR system with the following cycles: 1 cycle of 10 min at 50 °C for the reverse transcription reaction, 1 cycle of 1 min at 95 °C for polymerase activation, 40 cycles consisting of 15 s at 95 °C for denaturation, and 1 min at 60 °C for annealing. The changes in relative gene fold were calculated by the comparative C_t_ method (2^−ΔΔCt^) and the target genes’ expression was normalized to the β-actin. The sequences of the primers have been listed in Table 1.

### 2.10. In Vitro BBB-GBM Model 

As the IONPs for GBM therapy would be required to first pass the BBB, a brain endothelial cell–GBM cell co-culture model was established to assess the permeability and anticancer effects of the IONP formulations. For this study, bEnd.3 cells were plated on the apical side of a porous polyethylene terephthalate (PET) membrane (cell culture inserts, pore size: 3.0 μm, BD Bioscience, Franklin Lakes, NJ, USA). Once confluent bEnd.3 monolayers were obtained (typically in 7 days), U251 cells were cultured in the basolateral side of the well plates to assess both permeability and pharmacological responses. Free salinomycin (1 µg/mL) or Sali-PEI-PEG-IONPs was added to the apical media compartment of the insert along with a 70 kDa fluorescein isothiocyanate-dextran (FDX70000) permeability marker. The cells were then incubated at 37 °C for 6 h in both the presence and absence of a static magnetic field. Thereafter, the apical media and the inserts were withdrawn and the U251 cells with the basolateral cell culture media were incubated for an additional 24 h, after which the basolateral media was collected to determine IONPs (ferrozine assay) and the cell viability (MTT assay) was examined. To assess permeability following transient disruption of the bEnd.3 monolayer through the hyperosmotic condition, the bEnd.3 monolayers were pretreated with DMEM containing 1.4 M mannitol for 2 h, after which the monolayers were washed with PBS and then placed in 6-well plates containing U251 cells for permeability and cytotoxicity studies, as described above.

### 2.11. Statistical Analysis

All quantitative results were acquired from triplicate samples and data were expressed as the mean ± standard deviation (SD). Statistical analysis was performed using analysis of variance (ANOVA) and *p* < 0.05 was considered as the criterion of significance, as previously reported [37,38,39].

## 3. Results and Discussion

### 3.1. Characterization of IONPs 

**Figure 1:**
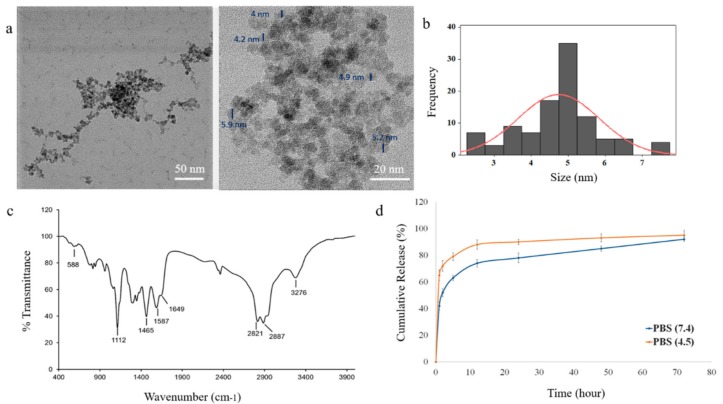
Characterization of polyethylenimine polyethylene glycol iron oxide nanoparticles (PEI-PEG-IONPs) and release of salinomycin from salinomycin-loaded (Sali)-PEI-PEG-IONPs: (**a**) transmission electron microscopy (TEM) images of PEI-PEG-IONPs, with core size measurement of the nanoparticles; (**b**) size distribution histogram; (**c**) Fourier transform infrared (FTIR) spectrum; (**d**) release of salinomycin from the nanoparticles in pH 7.4 (physiological pH) and pH 4.5 (pH of acidic intracellular compartments such as endosomes).

The TEM image of the PEI-PEG-IONPs indicates the nanoparticles have a quasi-spherical morphology and a core size of 4.76 ± 0.7 nm (Figure 1a,b). The PEI-PEG-IONPs were also characterized using energy-dispersive X-ray spectroscopy for elemental analysis (Figure S1). In addition, the hydrodynamic diameter (D_H_) and zeta potential (ζ) of the PEI-PEG-IONPs were 84.1 ± 14 nm (polydispersity index: 0.132) (Figure S2) and +27.14 mV, respectively. The FTIR spectrum of the nanoparticles is depicted in Figure 1c. The siloxane shell was characterized by the Si-O-Si stretching band at 991 cm^−1^. Symmetric and asymmetric C-H stretching (of propyl group) bands, as well as N-H bending (from free amine groups), were observed at 2821, 2887, and 1587 cm^−1^, respectively. The strong peak at 1112 cm^−1^ corresponds to the C-O-C stretching band of the conjugated PEG. The carbonyl stretching band from the amide linkage was also observed at 1649 cm^−1^ and the Fe-O-Fe stretching of the core was found at 588 cm^−1^. Magnetic and further physicochemical characterizations of the IONPs were previously reported elsewhere [33,40].

### 3.2. Characterization of the Sali-IONPs

The efficiency of salinomycin loading on the PEI-PEG-IONPs was calculated to be 3.45% ± 0.01% (*w*/*w*), based on the initial amount of salinomycin (30 µg/mL) used for ionic adsorption onto the nanoparticles and the total amount of salinomycin associated with the nanoparticles after washing. Loading of salinomycin decreased the ζ of the PEI-PEG-IONPs from +27.14 mV to +0.8 mV, indicative of an electrostatic interaction between the amine groups of PEI and the carboxyl groups of salinomycin. The release profile of salinomycin from the nanoparticles is illustrated in Figure 1d. The nanoparticles showed a burst release of salinomycin, with 44% ± 3% release within the initial hours, followed by a more sustained release over 72 h for the remaining coated drug. In addition, the acidic microenvironment accelerated the release of salinomycin from the nanoparticles up to 66% ± 5% within the initial hours, indicating the capability of an accelerated drug release once the nanoparticles enter the acidic tumor microenvironment or acidic intracellular compartments, such as endosomes [41,42]. The total amount of salinomycin released from the nanoparticles was determined to be 1030 ± 97 ng/mL. The initial burst release of the salinomycin observed in the present study is likely related to adsorption of the drug on the exterior regions of the polymer coating on the IONPs [43,44], as well as the weak electrostatic forces between the drug and the coating polymer. This study is the first to report the application of metal oxide nanoparticles for delivery of salinomycin and their potential applications for GBM chemotherapy. However, the salinomycin release profile obtained in the present study using IONPs is similar to those previously reported with polymer-based nanoparticle systems of salinomycin. For example, Chen et al. [45] developed lipid-polymer nanoparticles as a drug carrier of salinomycin for osteosarcoma treatment, with an initial burst release of ~60% and a cumulative drug release of ~80% in 72 h. Similarly, salinomycin-loaded PEGylated poly(lactic-co-glycolic acid) nanoparticles demonstrated a fast release of salinomycin (ca. 50%) in the initial 24 h, reaching the cumulative release of ~65% in the following 48 h [46]. 

Transient disruption of the BBB has been suggested as an effective approach to enhance the delivery of therapeutics across the BBB and treat malignant gliomas in clinical studies [47,48,49]. In this context, it should be noted that maximal disruption of the BBB after administration of mannitol, the most common hyperosmotic transient disruption agent, has been reported to occur within 5 min and lasts about 20–30 min in animal studies [50]. Therefore, the initial burst release of salinomycin from the nanoparticles, which have already been drawn to the target site using an external magnet, can increase the chance of the drug entering the brain within the optimum time frame of the BBB disruption. Moreover, the drug-loaded IONPs are expected to enter the brain within this time frame using both transient disruption of the BBB and magnetic targeting. Thus, a therapeutic concentration of the drug can be delivered to the tumor cells via the nanoparticles, even with an initial burst release of salinomycin. Chertok et al. [25] also reported that IONPs entered the brain within 1 h post-injection using magnetic targeting, even without transient disruption of the BBB. 

### 3.3. Biocompatibility of the IONPs

**Figure 2:**
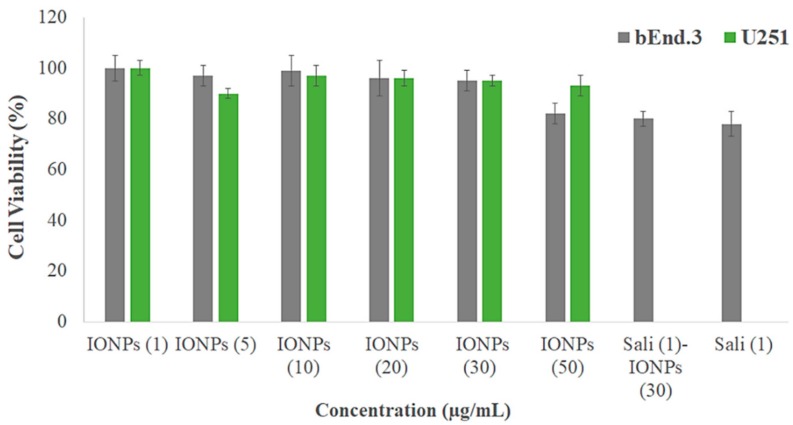
Biocompatibility of different concentrations of PEI-PEG-IONPs (termed IONPs) on mouse brain-derived microvessel endothelial (bEnd.3) and U251 cell lines after 48 h treatment using MTT assay (*n* = 5). The *Y*-axis represents cell viability compared to the control.

The biocompatibility of the PEI-PEG-IONPs on U251 and bEnd.3 was evaluated. The PEI-PEG-IONPs at a concentration ranging from 0.25 to 50 µg/mL did not show any cytotoxicity, *per se*, on U251 (Figure 2). In addition, their biocompatibility up to 320 µg/mL on HepG2 was previously reported [33]. Likewise, the PEI-PEG-IONPs at a concentration of 0.25 to 30 µg/mL did not show cytotoxicity on bEnd.3, albeit a minor reduction in cell viability was observed at the concentration of 50 µg/mL (Figure 2). Therefore, the concentration of 30 µg/mL of PEI-PEG-IONPs was selected for the next steps of this study. Although a minor reduction (ca. 20%) in cell viability was observed in the bEnd.3 cells with either salinomycin alone or Sali-IONPs compared to IONPs alone (30 µg/mL), this is merely attributed to the intrinsic feature of salinomycin, while the IONPs did not show any cytotoxicity at the same concentration (30 µg/mL) on bEnd.3 cells.

In clinical practice, iron oxide nanoparticles generally exhibit a desired biocompatibility profile and they are mainly captured by the reticuloendothelial system (RES), whereby the iron is incorporated into the body’s iron cycle [7,51]. Coating the iron oxide nanoparticles with hydrophilic macromolecules such as PEG reduces the non-specific protein adsorption on the nanoparticles and avoids their recognition and clearance by the RES, which ultimately leads to an extended circulation time of the nanoparticles, as well as enhanced accumulation in the brain tumor [52,53].

### 3.4. Cellular Uptake of the IONPs

**Figure 3:**
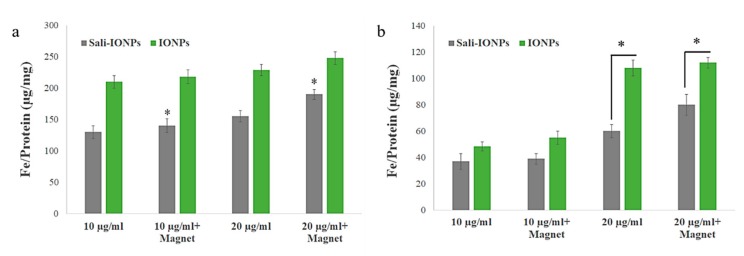
Uptake of IONPs and Sali-IONPs by (**a**) bEnd.3 and (**b**) U251 after 4 h treatment. Note: * indicates a significant difference at *p* < 0.05. Data was presented as mean ± standard deviation (SD); *n* = 3.

**Figure 4:**
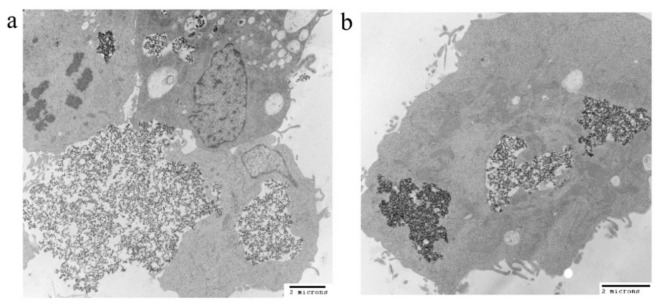
TEM images of (**a**) IONP and (**b**) Sali-IONP uptake by U251 cells after 4 h of the treatment.

The uptakes of both PEI-PEG-IONPs and Sali-PEI-PEG-IONPs in bEnd.3 (Figure 3a) and U251 (Figure 3b) were examined. The nanoparticle uptake in the cells was concentration-dependent, while application of an external magnetic field resulted in slight increases in IONP uptake, regardless of the formulation (i.e., PEI-PEG-IONPs or Sali-PEI-PEG-IONPs). Generally, the bEnd.3 had greater uptake of the nanoparticles than that of U251, and the higher uptake of PEI-PEG-IONPs can be attributed to the more positive charge on the bare nanoparticles compared to the Sali-PEI-PEG-IONPs. This finding is consistent with our previous studies reporting a higher uptake of positively charged IONPs in bEnd.3 cells, astrocytes, and neurons compared to negatively charged IONPs, owing to the electrostatic interactions between positively charged nanoparticles and the negatively charged plasma membrane of the cells [34]. Likewise, higher cellular uptake of positively charged iron oxide nanoparticles was reported in various mammalian cell lines in comparison to the negatively charged and neutral formulations [54]. Furthermore, the uptake of the nanoparticles appeared to be primarily through endocytosis, as TEM images clearly showed the distribution of the nanoparticles to be along the outside plasma membrane and within intracellular vesicles (i.e., endosomes and lysosomes) (Figure 4a,b, uptake by U251 cells; and Figure S3, uptake by bEnd.3 cells).

### 3.5. Cytotoxicity of Sali-IONPs on Cancer Cell

**Figure 5:**
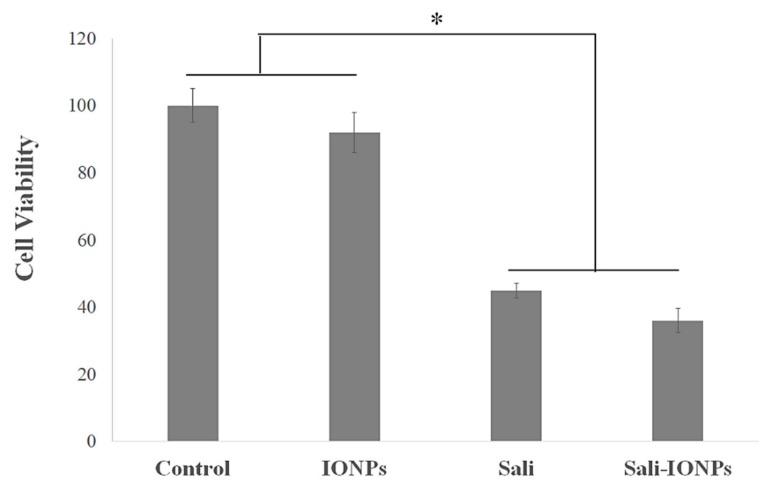
Cytotoxicity evaluation of salinomycin and Sali-IONPs on U251 after 48 h treatment. Note: * indicates a significant difference compared to the control group at *p* <0.05. Data was presented as mean ± SD; *n* = 6.

**Figure 6:**
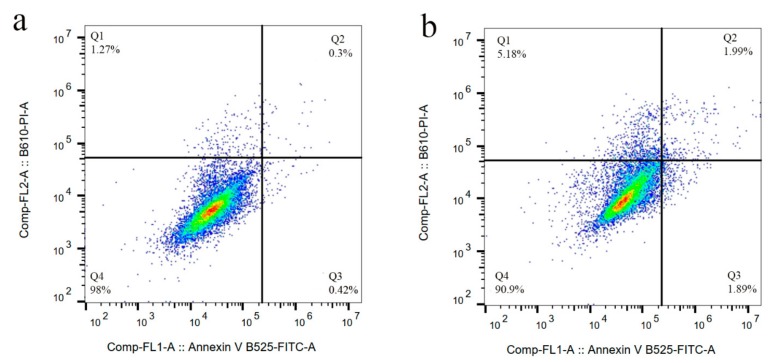

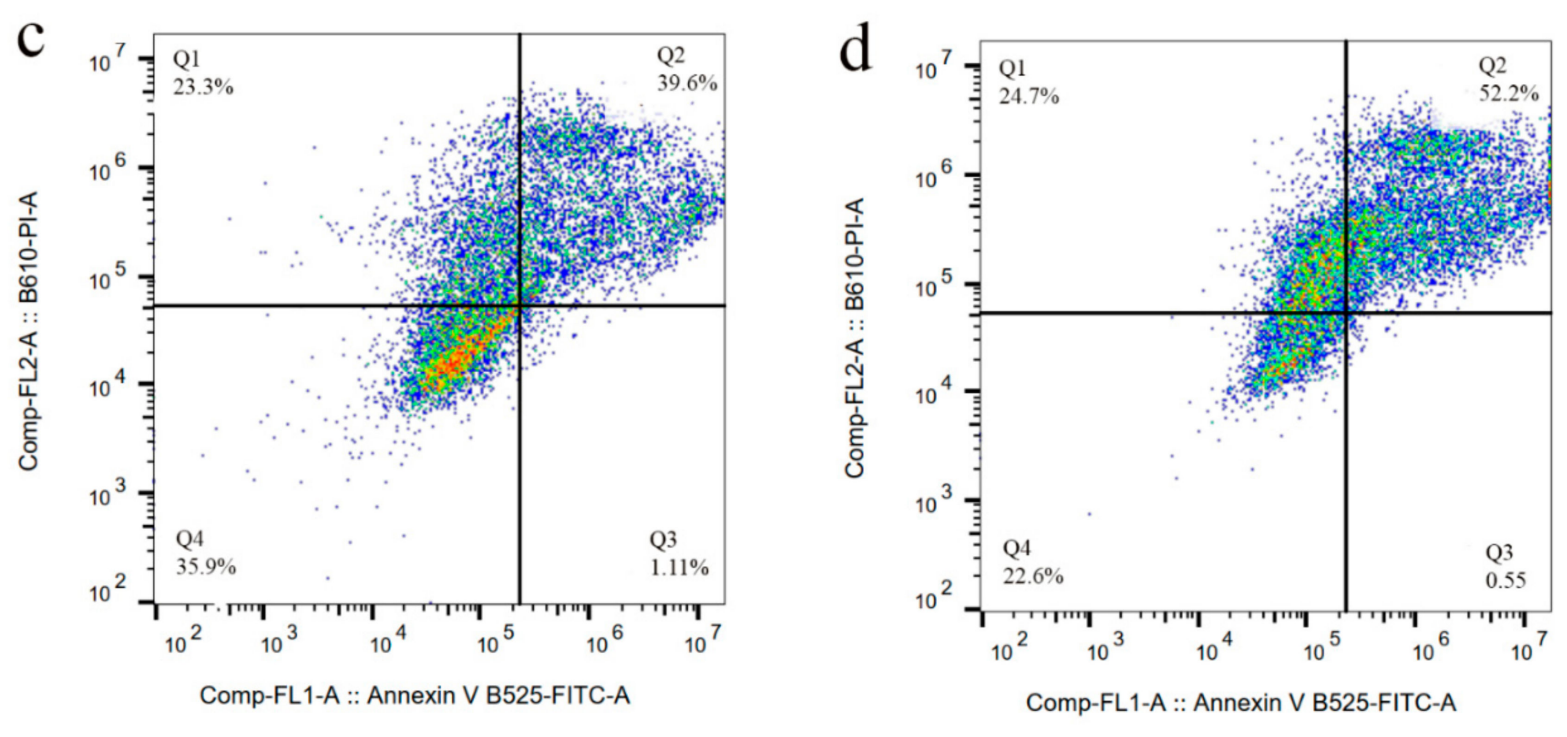
Cell apoptosis/necrosis of U251 upon treatment, stained with FITC-Annexin V and PI: (**a**) control, (**b**) IONPs (**c**) salinomycin, and (**d**) Sali-IONPs, with (Q4) live, (Q3) early apoptotic, (Q2) late apoptotic, and (Q1) necrotic cells.

**Figure 7:**
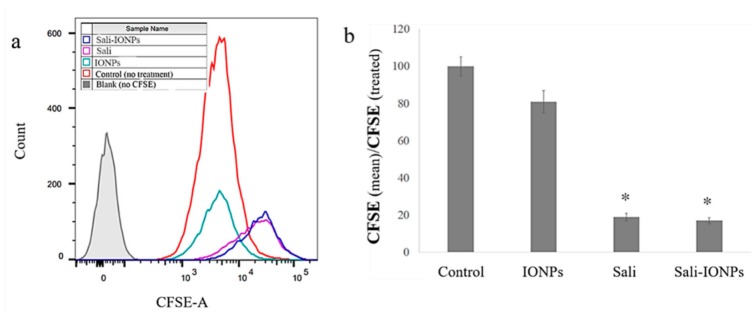
Cell proliferation analysis of carboxyfluorescein succinimidyl ester (CFSE)-labelled U251 upon treatment with IONPs, salinomycin, and Sali-IONPs: (**a**) CFSE flow cytometry graph and (**b**) the relative cell proliferation inhibition by mean CFSE_control_/mean CFSE_treated_. Note: * indicates a significant difference compared to the control group at *p* < 0.05.

We previously studied the cytotoxicity effects of salinomycin on U251 human glioblastoma cell line [31]. Herein, the cytotoxicity of the Sali-PEI-PEG-IONPs on U251 was investigated in comparison to that of free salinomycin. Based on the MTT viability studies (Figure 5), while the PEI-PEG-IONPs themselves did not show cytotoxic effects on U251, both treatments with free salinomycin and Sali-PEI-PEG-IONPs (1 µg/mL of salinomycin) significantly decreased the cell viability to 45% ± 2.2% and 36% ± 3.5%, respectively. Correspondingly, both salinomycin and Sali-PEI-PEG-IONPs were found to be equally effective in inducing apoptosis and necrosis in treated U251 cells, and a diminution of the cell viability to 36% and 22.6%, respectively, was observed (Figure 6). Moreover, salinomycin and Sali-PEI-PEG-IONP treatments could significantly inhibit U251 cell proliferation (Figure 7). A similar cytotoxic and anti-proliferative response to salinomycin has been reported in other types of cancer cells, such as pancreatic [55], leukemia [56], prostate [57], lung [58], and ovarian [59] cancer cells.

**Figure 8:**
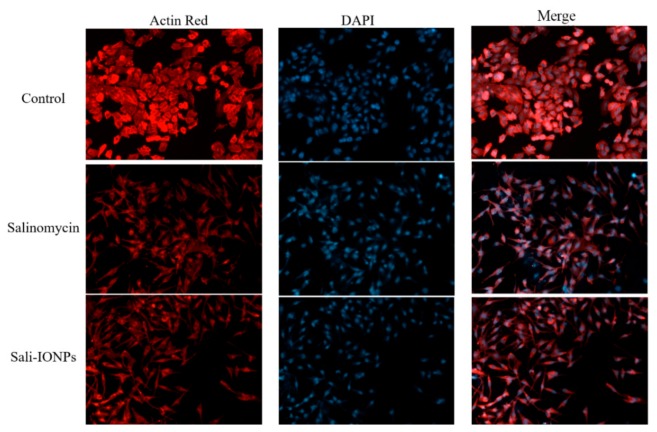
Fluorescence microscopy images of U251 treated with either salinomycin or Sali-IONPs after 48 h of the treatment. Red and blue fluorescence colors represent Alexa Fluor@ 488 phalloidin-stained F-actin and DAPI-stained cell nuclei, respectively.

The cell morphology was also examined by fluorescence microscopy, as shown in Figure 8. In addition to decreasing the cell number, both salinomycin and Sali-PEI-PEG-IONPs induced notable morphological changes in U251 cells following exposure. While the normal cells demonstrated a typical cuboidal morphology of U251, the actin cytoskeleton was changed to a shrunken and spindle-like structure upon treatment with either salinomycin or Sali-PEI-PEG-IONPs. Since the dynamic remodeling of the actin cytoskeleton is essential for cell migration, salinomycin can inhibit cell migration through a notable loss of actin stress fibers. Such responses to salinomycin have been reported in both pancreatic and liver cancer cells [55]. 

### 3.6. ROS Generation

**Figure 9:**
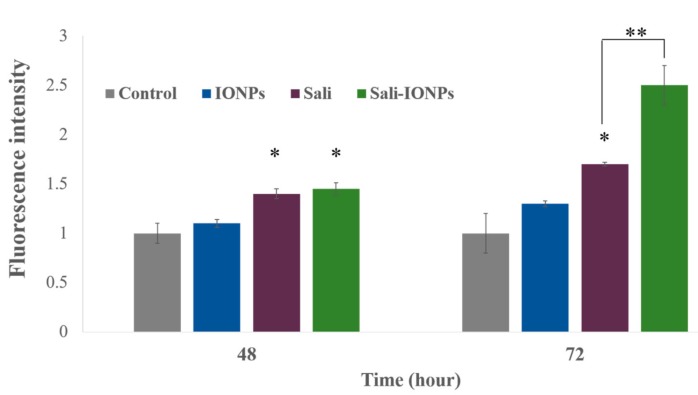
Reactive oxygen species (ROS) generation in U251 treated with either IONPs, salinomycin, or Sali-IONPs at different time-points. Note: * indicates a significant difference compared to the control group at *p* < 0.05. Data was presented as mean ± SD; *n* = 5.

Salinomycin-mediated ROS generation is known as a determining event committing the cancer cells to apoptotic death [57]. Increased formation of ROS was observed in the present study following exposure of U251 cells to salinomycin or Sali-PEI-PEG-IONPs (Figure 9). Both salinomycin and Sali-PEI-PEG-IONPs were effective in ROS generation in U251 cells after 48 and 72 h of the treatment, indicating the capability of inducing ROS-mediated apoptotic cell death. In this context, triggered ROS-mediated DNA damage has been suggested as a *de facto* mechanism of salinomycin-induced cell growth inhibition in human glioma cells [60]. Xipell et al. [61] also reported that salinomycin could trigger ROS generation in various glioma cell lines. As mentioned before, salinomycin is susceptible to multiple efflux transporters such as P-gp that restrict its cellular uptake and therapeutic efficacy [32], while the expression of both P-gp and multidrug resistance mutation (MDR1) in U251 GBM cells has been reported [62]. When nanoparticles are utilized as carriers for drugs that are susceptible to efflux transporters, their payloads are no longer substrate for the transporters due to masking effect by the nanoparticles, which can enhance the drug uptake by the cells through endocytosis of the drug-loaded nanoparticles [7]. Moreover, generation of intracellular ROS upon treatment with IONPs has widely been reported [63]. Although the PEG coating significantly increased the biocompatibility of IONPs in this study, meaning the ROS generation in U251 at 72 h for IONPs was not significant, the Sali-PEG-PEI-IONPs are likely to be more effective than free Sali in ROS generation due to the synergistic effect of PEG-PEI-IOPNs and Sali.

### 3.7. Quantitative RT-PCR

**Figure 10:**
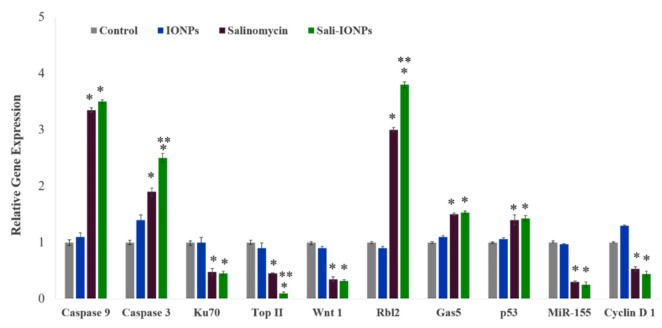
Relative gene expression of U251 cell treated with either IONPs, salinomycin, or Sali-IONPs after 48 h of the treatment. Note: * and ** indicate a significant difference compared to the control and salinomycin groups, respectively, at *p* < 0.05. Data was presented as mean ± SD; *n* = 5.

To elucidate the anticancer mechanisms of salinomycin and Sali-PEI-PEG-IONP treatments on U251, a series of genes was selected for investigation based on our previous *in vitro* studies (Figure 10). Activation of caspases and release of cytochrome c have been reported to be involved in salinomycin’s anticancer mechanisms [29]. Generally, caspases are triggered in a sequential manner, in which the activation of caspase 12 triggers activation of caspase 9 and the subsequent “effector”, caspase 3 [64]. The cell treatment with Sali-PEI-PEG-IONPs elevated caspase 9 and caspase 3 expression by 3.5 ± 0.2 and 2.5 ± 0.3-fold, respectively. Salinomycin’s effect on triggering caspase-dependent apoptosis by elevating the intracellular ROS level in prostate cancer cells has also been reported [57]. 

Ku70, a DNA-dependent protein kinase, is involved in the repair of DNA double-strand breaks and has been known as a survival factor in some cancer cells [65,66]. Sali-PEI-PEG-IONPs treatment could significantly reduce Ku70 expression by 0.48% ± 0.4%. Ku70 has been introduced as a primary resistance factor, whose knockdown could significantly chemo-sensitize gemcitabine-induced cell death and inhibit cell proliferation in pancreatic cancer cells [65]. Topoisomerase IIα (Top II) is another nuclear key enzyme in DNA replication, considered as the molecular target for some anticancer drugs (Top II inhibitors), such as etoposide and doxorubicin [67]. We found that both salinomycin and Sali-PEI-PEG-IONPs treatments could notably reduce Top II expression in GBM cells, while Top II expression has been found to be associated with high proliferation of cancer cells [68].

The Wnt signaling pathway is important in regulating stem cell self-renewal, and has been implicated in the pathogenesis of various cancers [69]. Furthermore, the Wnt signaling plays a critical role in malignant transformation and tumor progression in gliomas, while the therapeutic strategies aimed at silencing Wnt expression in glioma cells have shown a decreased capacity for intracranial tumor formation *in vivo* [70,71]. In this study, Wnt1 expression in U251 was significantly attenuated upon treatment with either salinomycin or Sali-PEI-PEG-IONPs. Inhibitory effects of salinomycin on Wnt signaling and apoptosis induction in lymphocytic leukemia cells [71], breast cancer cells [72], and gastric cancer stem cells [73] have previously been reported. 

Rbl2 belongs to the retinoblastoma (Rb) family that are considered as tumor suppressors. The Rb can inhibit cell cycle progression through disabling the E2F family of cell-cycle-promoting transcription factors [74]. Treatment with salinomycin and Sali-PEI-PEG-IONPs augmented Rbl2 expression by 2.7 ± 0.08 and 3.8 ± 0.1-fold, respectively, resulting in inhibition of the cell cycle progression. The long non-coding RNA (lncRNA) growth-arrest-specific 5 (Gas5) is another tumor-suppressor gene that is downregulated in several cancers, such as glioma, gastric, pancreatic, breast, prostate, lung, and colorectal cancers [75,76]. Introduction of Gas5 has been found to suppress tumor malignancy by downregulating miR-222 in GBM cell lines [77]. In the present study, salinomycin (both free salinomycin and Sali-PEI-PEG-IONPs formulations) significantly upregulated Gas5 expression in U251 cells. To the best of our knowledge, this is the first report of such changes in Gas5 with salinomycin exposure. 

Treatment of U251 cells with salinomycin and Sali-PEI-PEG-IONPs upregulated p53 expression in U251 GBM cells. The tumor suppressor protein p53 is the most common genetic alteration in human cancers, affecting about 50% of all tumor types [78]. Qin et al. [79] found that salinomycin could induce programmed necrosis via ROS-p53-cyclophilin-D signaling in U87 GBM cells. In this context, increased ROS caused unphosphorylated p53 to migrate into the mitochondrial matrix, where it bound to cyclophilin D (Cyp-D), forming a p53-CypD complex. It was reported that this complex can stimulate the opening of mitochondrial permeability transition pores, leading to a loss in the mitochondrial membrane potential and the release of cytochrome c, which ultimately leads to necrosis [80]. 

MiR-155 is a prominent oncogenic microRNA that regulates genes involved in immunity- and cancer-related pathways. MiR-155 is overexpressed in a variety of malignant tumors, whose mechanism of effect is accredited to a blockade of caspase-3 activity [81]. In this study, we reported for the first time that both salinomycin and Sali-PEI-PEG-IONPs could significantly attenuate MiR-155 expression in GBM cells. 

Cyclin D1 is a key regulator protein for the G1-S cell cycle phase transition and cell proliferation, whose overexpression is predominantly associated with human tumorigenesis and cellular metastases in a variety of cancers, including GBM [82,83,84]. Salinomycin and Sali-PEI-PEG-IONP-treated cells demonstrated a downregulation of Cyclin D1 that is consistent with salinomycin’s effect in downregulating cyclin D1 in ovarian [85], prostate, and breast [86] cancer cells. 

### 3.8. Evaluation of Sali-IONPs in BBB-GBM Co-Culture Model 

**Figure 11:**
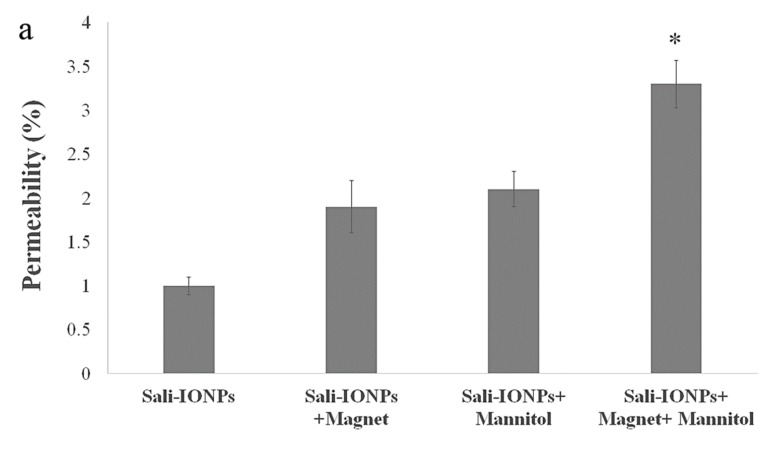

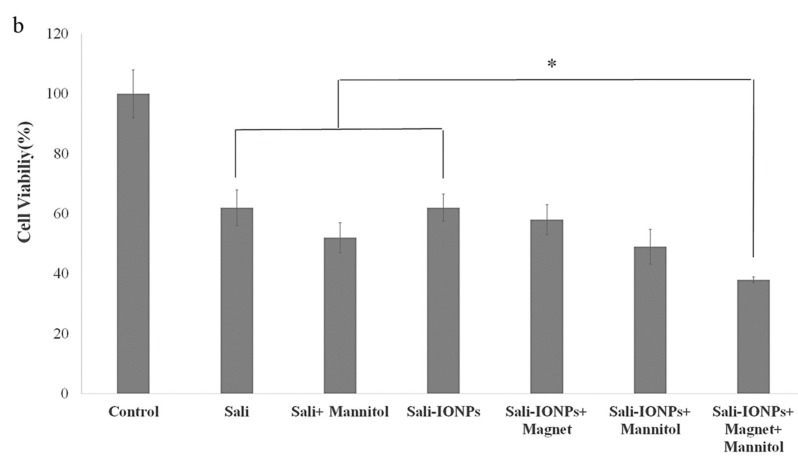
Evaluation of anticancer efficacy of Sali-IONPs compared to the free salinomycin on a BBB-brain tumor model *in vitro*: (**a**) Sali-IONPs permeability across the bEnd.3 monolayer with or without magnet and mannitol; (**b**) cytotoxicity of each formulation on U251 cells after penetrating the bEnd.3 monolayer. Note: * indicates a significant difference at *p* < 0.05 with the other treated groups. Data was presented as mean ± SD; *n* = 3.

As the potential treatments of GBM must be able to reach therapeutically relevant levels in the brain, the Sali-PEI-PEG-IONPs were examined using an *in vitro* BBB-GBM co-culture model. For this study, bEnd.3 cell monolayers were grown on Transwell inserts and placed in 6-well plates containing U251 cells for the study of the permeability and anticancer efficacy of the Sali-PEI-PEG-IONPs. Under normal conditions, Sali-PEI-PEG-IONPs had limited penetration across the bEnd.3 monolayers (1.0% ± 0.1% over 6 h). Permeability of the Sali-PEI-PEG-IONPs could be increased by either application of an external magnetic field (1.9% ± 0.3%) or by altering bEnd.3 monolayer integrity with hyperosmotic mannitol solution (2.1% ± 0.1%) (Figure 11a). Similarly, the FDX permeability marker showed permeability of 6.2% ± 0.4%, due to the smaller size than that of IONPs, which increased to 11.6% ± 0.3% with the administration of mannitol. While either the application of an external magnetic field to intact endothelial monolayers or application of a transient disruption agent increased the permeability of Sali-PEI-PEG-IONPs, the resulting effect on cytotoxicity to U251 cells was similar to that of salinomycin alone (Figure 11b). However, combining an external magnetic field with transient disruption of the endothelial monolayer resulted in even greater increases in Sali-PEI-PEG-IONP permeability (3.2% ± 0.1%), and as a result improved cytotoxicity in U251 cells (cell viability 62% ± 4% for free salinomycin vs. 38% ± 0.7% for Sali-PEI-PEG-IONPs + magnet + mannitol). With the enhanced permeability following application of mannitol and an external magnetic field, the cytotoxic responses in the BBB-GBM model were similar in magnitude to our initial cytotoxicity assessments in the U251 monocultures.

Generally, the physio-chemical properties of salinomycin suggest its limited BBB permeability and brain accumulation under normal conditions. Furthermore, the role of drug efflux transporters in restriction of the brain penetration of salinomycin has been reported *in vivo* [32]. In this study, the Sali-PEI-PEG-IONPs exhibited limited permeability under normal conditions. However, in the presence of an external magnetic field and transient disruption of the endothelial monolayer, significant increases in Sali-PEI-PEG-IONP permeability were observed. It is noteworthy to mention that utilizing an external magnetic field not only can enhance the passage of magnetic IONPs as a drug delivery system for chemotherapeutics across the BBB, but also can provide site-specific magnetic targeting to draw the nanoparticles to the site of action, regulating their systemic biodistribution and decreasing their systemic toxicity *in vivo* [25,87]. Taken together, it is suggested that utilizing transient disruption of the BBB in combination with an external magnetic field can potentially enhance the uptake of drug-loaded IONPs and augment the efficacy of GBM chemotherapy. 

## 4. Conclusions

In this study, Sali-PEI-PEG-IONPs were fabricated to facilitate salinomycin delivery to GBM tumor cells. Salinomycin was released from the PEI-PEG-IONPs over 4 days, with the capability of an accelerated initial release in the acidic microenvironments. The PEI-PEG-IONPs were found to be biocompatible in bEnd.3 and U251 cells, while both cell lines could efficiently uptake the nanoparticles. Moreover, the Sali-PEI-PEG-IONPs significantly induced ROS generation and apoptosis in U251 cells and inhibited their proliferation. The cell treatment with Sali-PEI-PEG-IONPs could efficaciously activate the caspase cascade and upregulated the tumor suppressors (i.e., p53, Rbl2, Gas5). Concomitantly, TopII, Ku70, CyclinD1, and Wnt1 were downregulated in the treated cells. Importantly, in an *in vitro* BBB-GBM co-culture model, the Sali-PEI-PEG-IONPs could augment salinomycin penetration through the bEnd.3 layer and provided more anticancer effects on U251 cells than that of free salinomycin in the presence of a magnetic field and mannitol. Therefore, the Sali-PEI-PEG-IONPs in combination with an external magnetic field and transient disruption of the BBB can be utilized as a new therapeutic platform to enhance salinomycin’s penetration into the brain and provide site-specific magnetic targeting for GBM chemotherapy. This approach not only increases salinomycin’s therapeutic efficiency, but also potentially diminishes the off-target exposure and toxicity.

## Figures and Tables

**Table 1:** Sequences of human primers.

Primer	Forward	Reverse
**TOP2**	ATTCCCAAACTCGATGATGC	CCCCATATTTGTCTCTCCCA
**Ku70**	CTGTCCAAGTTGGTCGCTTC	CTGCCCCTTAAACTGGTCAA
**p53**	TCTGAGTCAGGCCCTTCTGT	GTTCCGAGAGCTGAATGAGG
**Caspase 9**	CACGGCAGAAGTTCACATTG	AACAGGCAAGCAGCAAAGTT
**Caspase 3**	CTCTGGTTTTCGGTGGGTGT	CGCTTCCATGTATGATCTTTGGTT
**Cyclin D**	GTCCCACTCCTACGATACGC	CAGGGCCGTTGGGTAGAAAA
**Wnt1**	CAACAGCAGTGGCCGATGGTGG	CGGCCTGCCTCGTTGTTGTGAAG
**Rbl2**	GGTTCCCACTGAGTGATTACTGT	AGAAGCCTCCTATGCTCACG
**GAS5**	TGGTTCTGCTCCTGGTAACG	AGGATAACAGGTCTGCCTGC
**MIR 155**	AATCGTGATAGGGGTTTTTGCC	ATGTAGGAGTCAGTTGGAGGC
**β-actin**	AATGCCAGGGTACATGGTGG	AGGAAGGAAGGCTGGAAGAGTG

## Supplementary Materials

The following are available online at https://www.mdpi.com/2079-4991/10/3/477/s1. Figure S1: Energy-dispersive X-ray spectroscopy of PEI-PEG-IONPs. Figure S2: DLS graph of PEI-PEG-IONPs measured in DI water (pH 7.2). Figure S3: TEM images of IONP (top) and Sali-IONP (bottom) uptake by bEnd.3 cells after 4 h of the treatment.
