# Supplementary material for: Salinomycin-Loaded Iron Oxide Nanoparticles for Glioblastoma Therapy
Source: Nanomaterials (Basel). 2020 Mar 6;10(3):477. doi: 10.3390/nano10030477 (PMC7153627; doi:10.3390/nano10030477)
Supplement: Supplementary file 1 [file nanomaterials-10-00477-s001.pdf]

Supplementary Materials

Salinomycin-Loaded Iron Oxide Nanoparticles for Glioblastoma Therapy

Mohammad Norouzi [1,2], Vinith Yathindranath [2], James A. Thliveris [3] and Donald. W Miller [1,2,*,†]

[1] Department of Biomedical Engineering, University of Manitoba, Winnipeg, MB R3T 5V6 , Canada; norouzim@myumanitoba.ca

[2] Department of Pharmacology and Therapeutics, University of Manitoba, Winnipeg, MB R3E 0Z3, Canada; Vinith.Yathindranath@umanitoba.ca

[3] Department of Human Anatomy and Cell Science University of Manitoba, Winnipeg, MB R3E 0J9, Canada; james.thliveris@gmail.com

* Correspondence: donald.miller@umanitoba.ca; Tel.: +1-204-789-3278

† Current address: Department of Pharmacology & Therapeutics, University of Manitoba, A205 Chown Bldg., 753 McDermot Avenue, Winnipeg, MB R3E 0Z3, Canada

Received: 21 January 2020; Accepted: 03 March 2020; Published: date

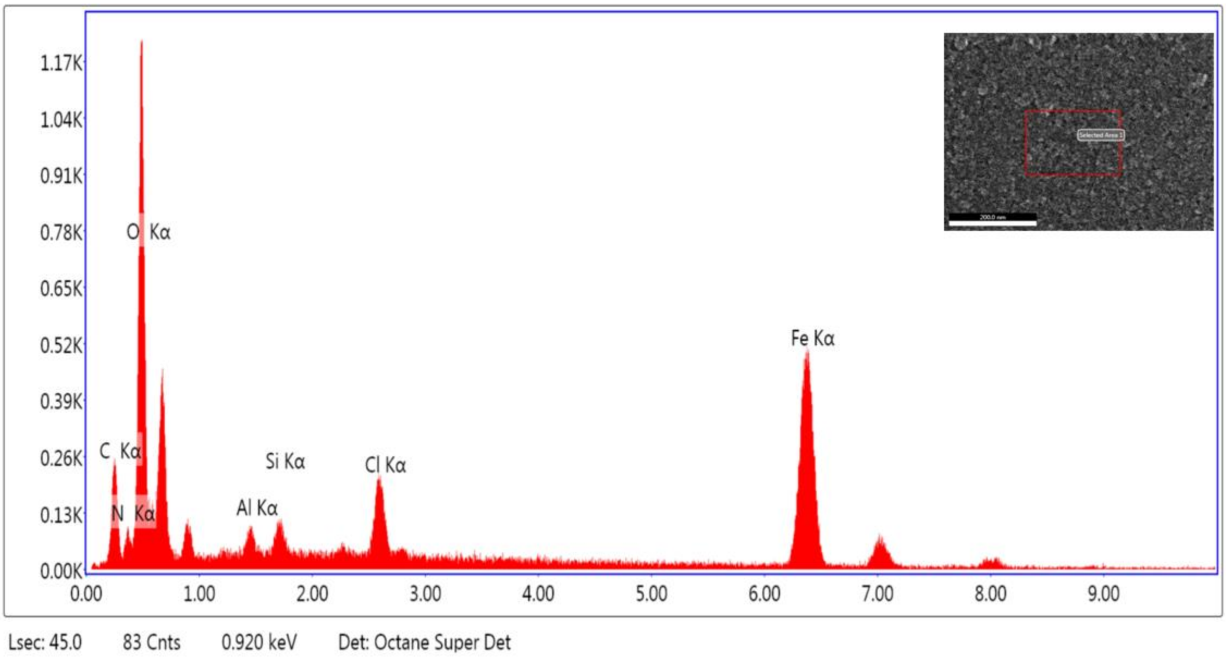

Element	Weight %	Atomic %	Error %
C K	10.48	22.30	11.46
N K	5.04	9.20	14.62
O K	24.73	39.49	8.11
AlK	0.69	0.65	22.42
SiK	0.95	0.86	15.47
ClK	3.47	2.50	10.13
FeK	54.63	24.99	3.72

Figure S1. Energy-dispersive X-ray spectroscopy of PEI-PEG-IONPs.

ionp-peg-pei2k_DIW (Combined)
May 16, 2018 14:00:50

Effective Diameter: 84.1 nm
Polydispersity: 0.132
Avg. Count Rate: 142.3 kcps
Baseline Index: 6.9/100.00%
Elapsed Time: 00:00:50

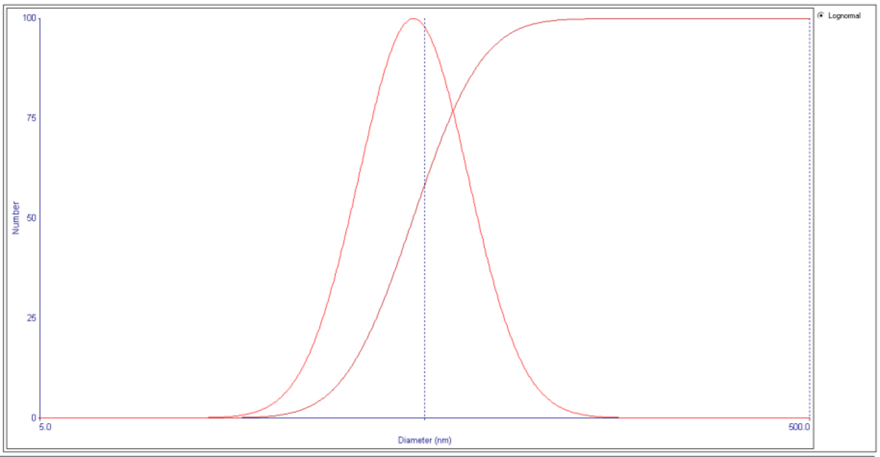

Run	Eff. Diam. (nm)	Half Width (nm)	Polydispersity	Baseline Index
1	80.8	30.8	0.145	1.5/100.00%
2	81.7	38.8	0.226	3.5/100.00%
3	85.8	25.8	0.091	7.0/100.00%
4	87.2	30.5	0.123	5.9/100.00%
5	82.6	31.3	0.144	9.9/100.00%
Mean	83.6	31.5	0.146	5.6/100.00%
Std. Error	1.2	2.1	0.022	1.4/ 0.00
Combined	84.1	30.5	0.132	6.9/100.00%

ionp-peg-pei2k_DIW (Combined)
May 16, 2018 14:00:50

Effective Diameter: 84.1 nm
Polydispersity: 0.132
Avg. Count Rate: 142.3 kcps
Baseline Index: 6.9/100.00%
Elapsed Time: 00:00:50

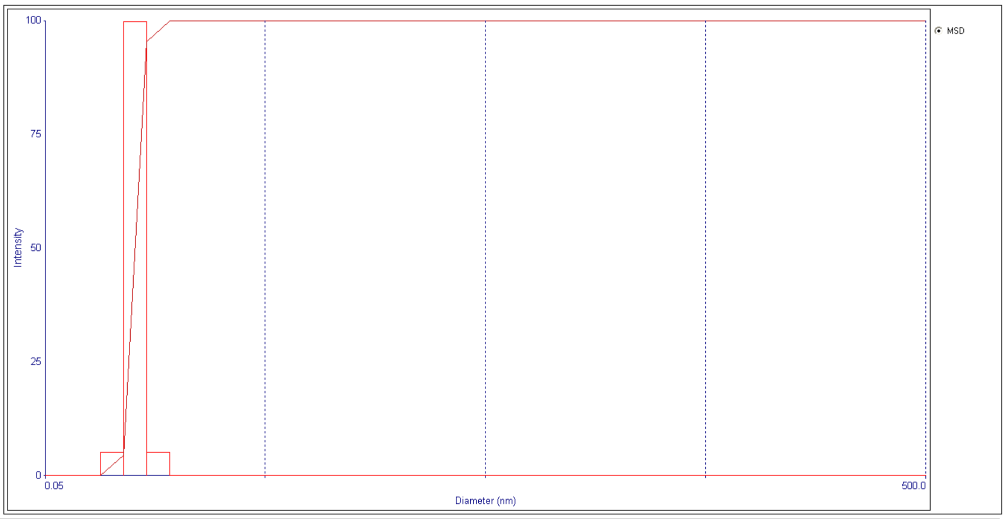

Run	Eff. Diam. (nm)	Half Width (nm)	Polydispersity	Baseline Index
1	80.8	30.8	0.145	1.5/100.00%
2	81.7	38.8	0.226	3.5/100.00%
3	85.8	25.8	0.091	7.0/100.00%
4	87.2	30.5	0.123	5.9/100.00%
5	82.6	31.3	0.144	9.9/100.00%
Mean	83.6	31.5	0.146	5.6/100.00%
Std. Error	1.2	2.1	0.022	1.4/ 0.00
Combined	84.1	30.5	0.132	6.9/100.00%

Figure S2. DLS graph of PEI-PEG-IONPs measured in D.I. water (pH 7.2).

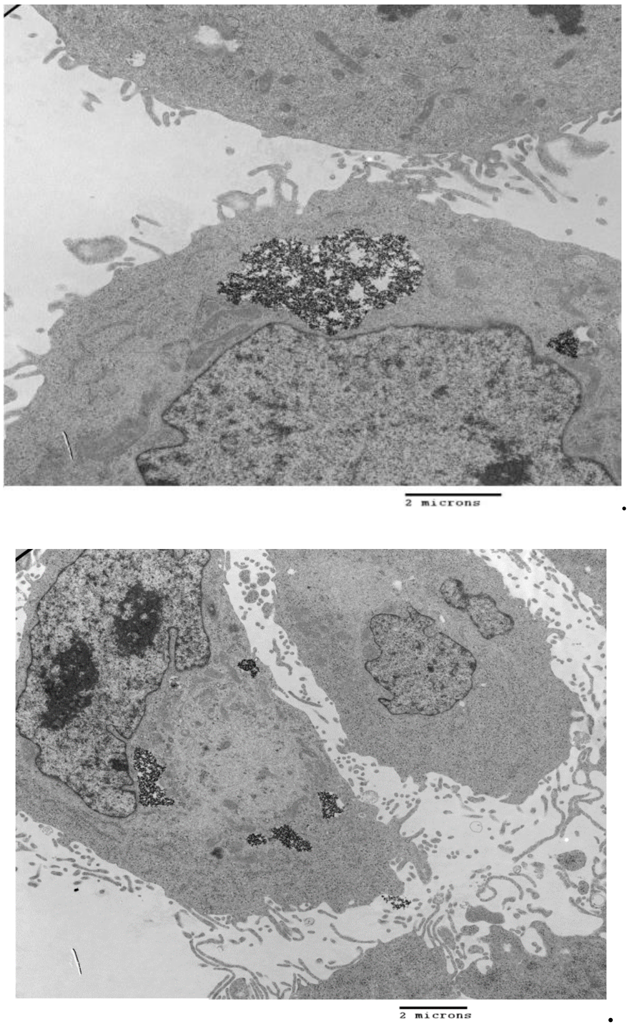

Figure S3. TEM images of IONPs (top) and Sali-IONPs (bottom) uptake by bEnd.3 cells after 4 h of the treatment.
